# Analysis of the clinical characteristics and surgical methods of high-grade squamous intraepithelial lesions of the cervix in postmenopausal women: A retrospective case study

**DOI:** 10.1097/MD.0000000000038657

**Published:** 2024-06-21

**Authors:** Xiaofeng Zhao, Rong Zhang, Shufang Song, Yu Wang, Xiaojie Mu

**Affiliations:** a Department of Obstetrics and Gynecology, The Affiliated Hospital of Inner Mongolia Medical University, Hohhot, China; b Maternal and Child Health Hospital of Inner Mongolia, Hohhot, China.

**Keywords:** cold knife conization, extra-fascial total hysterectomy, high-grade squamous intraepithelial lesions, loop electrosurgical excision procedure, postmenopausal women

## Abstract

The purpose of this study was to thoroughly evaluate the clinical features and surgical options for high-grade squamous intraepithelial lesions (HSIL) in postmenopausal women. A total of 308 patients diagnosed with HSIL through colposcopic cervical biopsy and endocervical curettage were included. Their clinical characteristics, surgical treatments, and postoperative pathology were analyzed. Key findings include: 1. Patients with positive preoperative thinprep cytologic test (TCT) results and postoperative pathology indicating HSIL or squamous cell carcinoma (≥HSIL) were significantly more frequent than those with negative preoperative TCT results (*P* < .05). 2. Univariate analysis indicated significant impacts of TCT, human papillomavirus (HPV) type, transformation zone (TZ) location, and surgical technique on postoperative pathology (*P* < .05). 3. Logistic regression analysis confirmed significant influences of TCT, HPV type, TZ location, and surgical method on postoperative pathology outcomes (*P* < .05), showing that each unit increase in TZ raised the probability of ≥HSIL in postoperative pathology by 49.7%. In surgical comparisons, cold knife conization (CKC) and extrafascial hysterectomy resulted in 8.379 and 4.427 times higher probabilities of ≥HSIL in postoperative pathology, respectively, compared to loop electrosurgical excision procedure (LEEP). 4. Surgical methods significantly influenced margin results (*P* < .05). After LEEP, 17.5% of cases had positive margins, compared to 9.4% after CKC, and 3.7% after extrafascial hysterectomy, indicating the highest rate of positive surgical margins occurred with LEEP. 1. Combined TCT and HPV screening is crucial for cervical cancer prevention, early detection, and management in postmenopausal women. Women with positive results for both TCT and HPV should undergo colposcopic cervical biopsy and endocervical curettage. 2. For patients with TZ3, CKC is the recommended surgical option. 3. CKC is the preferred treatment for postmenopausal women with HSIL, as it effectively diagnoses and treats the lesion, showing superior outcomes in managing postmenopausal HSIL.

## 1. Introduction

Cervical cancer is one of the malignant tumors that gravely endangers women’s health, and it is notably the only malignant tumor that can be eradicated through targeted tertiary screening measures.^[1]^ The process from high-risk human papillomavirus infection to the natural progression towards cervical cancer spans several decades.^[2]^ Human papillomavirus (HPV) infections are particularly prevalent in young women, especially those around the age of 20. The prevalence of HPV decreases significantly after the age of 25 to 30. However, a subsequent increase is observed in women between the ages of 50 and 60, most of whom are either perimenopausal or postmenopausal.^[3]^ Factors such as previous cervical screenings or the experience of becoming widowed may lead these women to mistakenly perceive themselves as being at a lower risk. This misperception tends to reduce their engagement in regular screening and lowers their awareness of potential diseases, subsequently increasing the incidence of cervical cancer.^[3]^ High-grade squamous intraepithelial lesions (HSIL) are detectable during cervical cancer screenings, and effectively treating HSIL can significantly reduce the incidence of cervical cancer.^[4]^ HSIL may naturally progress through 3 potential outcomes: regression, persistence, or progression. Importantly, HSIL carries an increased risk of developing into cervical cancer. Modeling studies indicate that untreated HSIL will evolve into invasive cancer in 15% to 23% of cases within an average of 13 years, with lifetime risk escalating to 40%.^[5]^ Moreover, postmenopausal women experience a reduction in estrogen levels, cervical atrophy, an inward shift of the squamous-columnar junction, and decreased sexual activity, all of which contribute to the absence of pronounced clinical symptoms and signs in the early stages, thus complicating the detection of cervical lesions in clinical settings.^[6]^ Furthermore, postmenopausal women often exhibit low cancer prevention awareness, conservative attitudes, and face economic constraints, which diminish their engagement in cervical screening. Their hesitance to seek prompt medical care, even after noticing abnormalities, typically results in delayed detection of cervical lesions in this demographic. In some instances, cervical cancer is detected at mid-to-late stages of invasion, leading to a poor prognosis and high mortality rates, which severely impact the physical and mental health of postmenopausal women.^[6]^ Additionally, the physiological characteristics of postmenopausal women differ markedly from those of sexually mature women due to atrophy of the lower genital tract, difficulty in fully exposing the cervical transformation zone, and thinning of the cervical epithelium, complicating the diagnosis and treatment of HSIL.^[7]^ This study discusses the clinical characteristics of postmenopausal women with HSIL and analyzes the postoperative pathological outcomes following various surgical interventions. The findings aim to provide appropriate recommendations for screening cervical lesions and selecting the optimal surgical approach for HSIL in postmenopausal women.

## 2. Materials and methods

### 2.1. General information

This study retrospectively collected data from 308 postmenopausal patients diagnosed with HSIL who were admitted to the Department of Gynecology at the Affiliated Hospital of Inner Mongolia Medical University between January 2020 and December 2022. The patients had an average age of 57.21 ± 6.37 years (range 40–79 years) and an average menopausal duration of 7.68 ± 6.52 years (range 1–39 years). Cases were categorized into 2 groups: those with negative results or low-grade squamous intraepithelial lesion (LSIL) were placed in the ≤LSIL group, and those with HSIL or squamous cell carcinoma (SCC) were placed in the ≥HSIL group. The inclusion criteria were as follows: 1. Menopausal status of one year or more. 2. Patients diagnosed with HSIL based on biopsy and endocervical curettage (ECC) pathology, who then underwent surgical treatment, with diagnoses confirmed by postoperative pathology. Exclusion criteria included: 1. Preoperative pathological diagnosis of cervical invasive carcinoma. 2. History of cervical lesion recurrence or prior treatment for cervical lesions. Data collected included Thinprep cytology test (TCT) and HPV results, colposcopic manifestations, colposcopic diagnoses, pathologic results following loop electrosurgical excision procedure (LEEP), cold knife conization (CKC), or extra-fascial total hysterectomy (ETH), and lesions at the margins of the incision. According to regulations in China, approval from an Ethics Committee was not required for this study as it did not involve traceable individual participant data, process sensitive personal data, nor require patient consent.

### 2.2. Research methods

#### 2.2.1. TCT detection

Cervical cytology was conducted using a liquid-based cytology test. Cytologic diagnoses were classified according to the Bethesda system classification endorsed by the International Cancer Society. The classifications include: negative for intraepithelial lesion or malignancy, atypical squamous cells of undetermined significance, atypical squamous cells cannot exclude high-grade squamous intraepithelial lesion, low-grade squamous intraepithelial lesion (LSIL), HSIL, SCC, atypical glandular cells, atypical glandular cells of undetermined significance, adenocarcinoma in situ, and adenocarcinoma.

#### 2.2.2. HPV test

HPV genotyping was conducted using a combination of in vitro polymerase chain reaction amplification and DNA reverse dot blot hybridization. The analysis was performed using the Line Gene 9660 fluorescent quantitative polymerase chain reaction instrument, manufactured by Hangzhou BIOER Technology Co., Ltd., China. The HPV genotyping detection kit, supplied by Guangdong Kaibio Biotechnology Co., Ltd., was employed to identify 17 high-risk HPV types (16, 18, 31, 33, 35, 39, 45, 51, 52, 53, 56, 58, 59, 66, 68, 73, 82, and 83). The procedures were executed strictly according to the kit’s instructions.

#### 2.2.3. Colposcope

Colposcopy was conducted in accordance with the Chinese expert consensus on colposcopy application. The terminology and diagnostic criteria employed were those recommended by the International Federation for Cervical Pathology and Colposcopy in 2011.^[8]^

#### 2.2.4. Loop electrosurgical excision procedure

The parameters that guide the extent and methodology of LEEP resection are based on the 2011 standards of the International Federation for Cervical Pathology and Colposcopy.^[8]^ For transformation zone 1 (TZ1), complete removal of the lesion is achieved. In cases involving transformation zone 2 (TZ2), resection extends beyond the minor transformation areas of the cervical epithelium. For transformation zone 3 (TZ3), the scope of resection is more extensive than in TZ1 and TZ2, covering areas of cervical epithelium that have diagnostic significance but are not observable through colposcopy.

#### 2.2.5. Cold knife conization of the cervix

CKC was employed as the initial treatment for most patients. The resection area was classified by type based on the transformation zone and the location of the squamous columnar epithelium. The length of the cervix to be resected was determined by the TZ type: 7 to 10 mm for TZ1, 10 to 15 mm for TZ2, and 15 to 25 mm for TZ3. Clinical procedures should also be tailored to the individual patient’s cervical length. The excised conization tissue was routinely subjected to pathological examination.^[9]^

#### 2.2.6. Extra-fascial total hysterectomy

A minority of patients chose ETH as their initial treatment due to several factors: severe atrophy of the lower reproductive tract, difficulty in achieving optimal surgical field exposure, limited surgical space, indistinct anatomical structures, suboptimal patient compliance, difficult follow-up conditions, and significant psychological stress.

#### 2.2.7. Pathological diagnosis

The diagnostic criteria for HSIL adhere to the 2014 WHO Classification of Tumors of Female Reproductive Organs.^[10]^ HSIL encompasses cervical intraepithelial neoplasia grades 2, 2 to 3, and 3. The definitive pathological diagnosis is established based on the highest level of pathology confirmed through cervical biopsy or surgical intervention. A positive resection margin for HSIL is characterized by pathological findings of HSIL within a distance of ≤3 mm from the excised margin of the cervical specimen post-surgery. This assessment includes both the endocervical margin (inner margin) and the exocervical vaginal margin (outer margin).

### 2.3. Statistical methods

In this study, SPSS 25.0 software was utilized for statistical analysis, including descriptive statistics, comparative analyses, and binary logistic regression. Frequency counts were used to represent data for comparative analyses, and a chi-square test was applied for cross-tabulations. For measurement data not following a normal distribution, quartiles were used to express the data [median (25th percentile, 75th percentile)]. The nonparametric Mann–Whitney U test was employed to compare differences between 2 groups, and the Kruskal–Wallis test was used for comparisons among multiple groups. Statistical significance was set at *P* < .05.

## 3. Results

### 3.1. Differences between preoperative TCT, HPV results, and postoperative pathology results

The relationship between preoperative TCT results, HPV status (positive or negative), and their combined effect (double positivity) with postoperative pathological outcomes was analyzed, as presented in Table 1. The analysis revealed that cases with positive preoperative TCT and postoperative pathology indicating high-grade squamous intraepithelial lesions (≥HSIL) were significantly more frequent compared to those with negative preoperative TCT (*P* < .05). However, the influence of HPV positivity or negativity on postoperative pathology outcomes was not statistically significant (*P* > .05). Importantly, the incidence of postoperative pathology indicating ≥HSIL was significantly higher in cases with both TCT and HPV positivity compared to those without double positivity (*P* < .05).

**Table 1:** Comparison of preoperative TCT and HPV results with postoperative pathological results.

Inspection items	Postoperative pathological		χ2	*P*
	≤LSIL (n = 115)	≥HSIL (n = 193)		
*TCT [n (%)]*			8.371	.004
Negative	46 (49.5)	47 (50.5)		
Positive	69 (32.1)	146 (67.9)		
*HPV [n (%)]*			0.398*	.528
Negative	1(16.7)	5(83.3)		
Positive	114 (37.7)	188 (62.3)		
*Double positive [n (%)]*			6.931	.008
No	47 (48.0)	51 (52.0)		
Yes	68 (32.4)	142 (67.6)		

HPV = human papillomavirus, HSIL = high-grade squamous intraepithelial lesions, LSIL = low-grade squamous intraepithelial lesion, TCT = thinprep cytology test.

* Indicates the application of a continuity correction.

### 3.2. Univariate analysis of the factors affecting postoperative pathological results

A comparison of the general data is presented in Table 2. The results indicate that the TCT, HPV type, transformation zone (TZ), and surgical method significantly impacted the postoperative pathology outcomes (*P* < .05). Conversely, factors such as age, duration of menopause, clinical symptoms, colposcopic visibility, squamous columnar junction, and colposcopic interpretation did not influence the postoperative pathological results (*P* > .05).

**Table 2:** Univariate analysis of factors affecting postoperative pathological results.

Influencing factors	Postoperative pathological				H/χ2	*P*
	Negative (n = 43)	LSIL (n = 72)	HSIL (n = 184)	SCC (n = 9)		
MA (years)	56 (52,61)	57.5 (52,62)	57 (53,61.7)	55 (53,59)	0.886H	.829
MT (years)	6 (2,10)	5.5 (2,10)	6 (2,12)	4 (2,6.5)	1.965H	.58
*CS [n (%)]*					5.163fχ2	.14
Negative	39 (14.1)	67 (24.3)	164 (59.4)	6 (2.2)		
Positive	4 (12.5)	5 (15.6)	20 (62.5)	3 (9.4)		
*TCT [n (%)]*					30.577χ2	.032
NILM	13 (14.3)	32 (35.2)	43 (47.3)	3 (3.3)		
ASC-US	19 (14.3)	26 (22.4)	69 (59.5)	2 (1.7)		
ASC-H	5 (20.8)	3 (12.5)	13 (54.2)	3 (12.5)		
LSIL	4 (11.8)	4 (11.8)	26 (76.5)	0 (0)		
HSIL	2 (5)	6 (15)	31 (77.5)	1 (2.5)		
SCC	0 (0)	0 (0)	1 (100)	0 (0)		
None	0 (0)	1 (50)	1 (50)	0 (0)		
*HPV [n (%)]*					17.968fχ2	.022
Negative	1 (16.7)	0 (0)	5 (83.3)	0 (0)		
16+	18 (16.7)	20 (18.5)	64 (59.3)	6 (5.6)		
16+ with other high-risk	5 (6.3)	16 (20.3)	57 (72.2)	1 (1.3)		
Other high-risk	19 (16.5)	36 (31.3)	58 (50.4)	2 (1.7)		
*CE [n (%)]*					5.606fχ2	.125
Adequacy	31 (17.6)	37 (21)	104 (59.1)	4 (2.3)		
Inadequate	12 (9.1)	35 (26.5)	80 (60.6)	5 (3.8)		
*SCJ [n (%)]*					7.750fχ2	.248
Fully visible	17 (19.3)	21 (23.9)	49 (55.7)	1 (1.1)		
Not fully visible	19 (15.2)	30 (24)	72 (57.6)	4 (3.2)		
Not visible	7 (7.4)	21 (22.1)	63 (66.3)	4 (4.2)		
*TZ [n (%)]*					13.674fχ2	.027
1	17 (22.1)	20 (26)	39 (50.6)	1 (1.3)		
2	17 (14)	33 (27.3)	68 (56.2)	3 (2.5)		
3	9 (8.2)	19 (17.3)	77 (70)	5 (4.5)		
*CR [n (%)]*					0.450fχ2	.950
HSIL	30 (13.3)	53 (23.5)	136 (60.2)	7 (3.1)		
LSIL	13 (15.9)	19 (23.2)	48 (58.5)	2 (2.4)		
*SP [n (%)]*					38.982fχ2	<.001
LEEP	14 (35)	18 (45)	8 (20)	0 (0)		
CKC	13 (8.2)	30 (18.9)	111 (69.8)	5 (3.1)		
ETH	16 (14.7)	24 (22)	65 (59.6)	4 (3.7)		

*Note*: χ2 means Chi-square test, f means Fisher exact test, and H means nonparametric Kruskal–Wallis test.

ASC-H = atypical squamous cells cannot exclude HSIL, ASC-US = atypical squamous cell of undetermined significance, CE = colposcopic exposure, CR = colposcope results, CKC = cold knife conization, CS = clinical symptoms, ETH = extra-fascial total hysterectomy, HPV = human papillomavirus, HSIL = high-grade squamous intraepithelial lesions, LEEP = loop electrosurgical excision procedure, LSIL = low-grade squamous intraepithelial lesion, MA = menopause age, MT = menopause time, NILM = negative for intraepithelial lesion or malignancy, SCC = squamous cell carcinoma, SCJ = squamocolumnar junction, SP = surgical procedures, TCT = thinprep cytology test, TZ = transformation zone.

### 3.3. Logistic regression analysis

Binary logistic regression analysis was performed to assess the impact of the TCT, HPV type, TZ, and surgical method on postoperative pathological outcomes. Given the small sample size and the varied categories of postoperative pathology, TCT, and HPV, combined variable assignments were utilized. These assignments for each variable are detailed in Table 3. Results of the logistic regression analysis, post-assignment, are presented in Table 4. The analysis indicated that TCT significantly influences postoperative pathology (*P* < .05). Similarly, HPV type was found to have a significant effect on postoperative pathology (*P* < .05). Additionally, the impact of TZ on postoperative pathology was significant (*P* < .05), with each unit increase in TZ associated with a 49.7% increase in the likelihood of postoperative pathology results indicating ≥HSIL. Moreover, the type of surgical procedure had a significant effect on postoperative pathology outcomes (*P* < .05). Specifically, compared to LEEP, the odds of obtaining ≥HSIL postoperative pathology results were 8.379 and 4.427 times higher for CKC and ETH, respectively.

**Table 3:** Variables and assignments.

Variables		Assignment
Dependent variable	Postoperative pathology	Negative, LSIL assigned 0. HSIL, CC assigned 1.
Independent variable	TCT	None and NILM assigned 0. ASC-US, ASC-H, LSIL assigned 1. HSIL, SCC assigned 2. (NILM is used as a reference)
	HPV	Negative is assigned 0. 16+, 16+ with other high-risk is assigned 1. Other high risk is assigned 2. (Negative as reference)
	TZ	1 assigned value 1. 2 assigned value 2. 3 is assigned 3.
	SP	LEEP assigned 0. CKC assigned 1. Extra-fascial hysterectomy assigned 2.

ASC-H: atypical squamous cells cannot exclude HSIL, ASC-US = atypical squamous cell of undetermined significance, HPV = human papillomavirus, HSIL: high-grade squamous intraepithelial lesions, LSIL = low-grade squamous intraepithelial lesion, TCT = thinprep cytology test, TZ = transformation zone, SCC = squamous cell carcinoma, SP = surgical procedures.

**Table 4:** Logistic regression analysis of factors influencing postoperative pathology.

Influencing factors	Results of calculation					OR (95%CI)
	B	Standard error	Walder	Freedom	*P*	
TCT (0)			7.537	2	.023	
TCT (1)	0.547	0.286	3.649	1	.056	1.728 (0.986–3.03)
TCT (2)	1.211	0.475	6.498	1	.011	3.358 (1.323–8.524)
HPV (0)			6.973	2	.031	/
HPV (1)	‐0.596	1.125	0.281	1	.596	0.551 (0.061–4.997)
HPV (2)	‐1.267	1.130	1.258	1	.262	0.282 (0.031–2.579)
TZ	0.404	0.185	4.777	1	.029	1.497 (1.043–2.15)
SP (0)			23.788	2	<.001	/
SP (1)	2.126	0.448	22.498	1	<.001	8.379 (3.481–20.168)
SP (2)	1.488	0.477	9.709	1	.002	4.427 (1.737–11.284)
Constant	‐1.534	1.246	1.516	1	.218	0.216

HPV = human papillomavirus, SP = surgical procedures, TCT = thinprep cytology test, TZ = transformation zone.

### 3.4. Comparison of postoperative pathological margins of different surgical methods

Table 5 presents a comparison of pathological margins observed following various surgical procedures. Positive margins were detected in 17.5% of cases post-LEEP, 9.4% post-CKC, and 3.7% post-ETH, indicating a statistically significant difference among the procedures (*P* < .05). Notably, the highest rate of positive pathological margins occurred following LEEP.

**Table 5:** Comparison of postoperative pathological margins of different surgical methods.

Variables		Negative margins (n = 288)	Positive margin (n = 20)	χ2	*P*
SP	LEEP [n (%)]	33 (82.5)	7(17.5)	7.660f	<.05
	CKC [n (%)]	144 (90.6)	15(9.4)		
	ETH [n (%)]	105 (96.3)	4(3.7)		

*Note*: f indicates Fisher exact test.

CKC = cold knife conization, ETH = extra-fascial total hysterectomy, LEEP = loop electrosurgical excision procedure, SP = surgical procedures.

## 4. Discussion

Annually, new cases of cervical cancer among individuals over 45 years old constitute approximately 70% of the total in China, highlighting postmenopausal women as a critical demographic for cervical cancer screening.^[11]^ Epidemiological studies reveal a bimodal incidence of cervical cancer, with the first peak occurring around the age of 40 and the second among older women aged 65 to 79.^[12]^ This underscores the importance of enhanced cervical cancer screening for postmenopausal women to effectively prevent the disease. According to the 2019 Risk-based Consensus Guidelines for the Management of Abnormalities and Precancerous Lesions in Cervical Cancer Screening, published by the American Society for Colposcopy and Cervical Pathology in 2020, excisional treatments such as LEEP, CKC, and laser conization are recommended for managing HSIL.^[13]^ Postmenopausal patients diagnosed with HSIL have access to various surgical options including CKC, LEEP, hysterectomy, endoscopically assisted cervical conization, large loop excision of the TZ, hysteroscopy, hysteroscopic cervical conization, and total hysterectomy.^[14]^ Characteristics unique to menopausal patients, such as a reduction in the number of squamous epithelial cell layers, diminished cell volume, and decreased cervical mesenchyme, result in a pale and atrophic appearance of the cervix. Additionally, the cervical columnar epithelium becomes atrophied, losing its typical villous structure. The vaginal portion of the cervix shortens, and the squamocolumnar junction moves inward into the cervical canal, making cervical lesions more likely to hide within the canal and challenging to detect.^[15]^ This study investigates the characteristics of cervical lesions and surgical approaches in postmenopausal women to analyze the clinical features of HSIL and identify optimal surgical strategies.

### 4.1. Clinical characteristics of HSIL in postmenopausal women

In postmenopausal patients, early clinical symptoms and signs of cervical intraepithelial lesions are often inconspicuous. Sole reliance on gynecological examinations can make definitive identification of these lesions challenging. Moreover, the presence or absence of clinical symptoms does not significantly impact postoperative pathological outcomes. This study corroborates findings by Hao,^[16]^ indicating that clinical symptoms do not significantly affect postoperative pathological findings (*P* > .05). This phenomenon may be attributed to decreased estrogen levels, age-related cervical atrophy, and the upward shift of the TZ in postmenopausal women. Consequently, postmenopausal women should remain vigilant about endogenous cervical lesions. ECC is essential to further confirm diagnoses.^[17]^ These findings underscore the importance of regular cervical cancer screening for postmenopausal women, emphasizing that it should not be overlooked due to the patient’s age at menopause, the prolonged duration of menopause, or the absence of clinical symptoms.

### 4.2. Impact of cervical cancer screening on postoperative pathologic results in postmenopausal women

With the rapid development of the social economy, improvements in living standards, and heightened healthcare awareness, coupled with the widespread adoption of cervical cancer screening, many precancerous cervical lesions in women of childbearing age are being detected early and treated promptly. This proactive approach effectively prevents the progression of cervical intraepithelial lesions, significantly reducing the incidence of cervical cancer. For postmenopausal women, a key focus of clinical practice is the early detection of cervical lesion characteristics and the selection of screening methods with a high cost-effectiveness ratio. Currently, the three-step principle of cervical cancer prevention—screening, colposcopy evaluation, and histological diagnosis—is the standard protocol in clinical settings for cervical cancer screening and diagnosis.^[18]^ Research has shown that combined screening using the TCT and HPV testing significantly enhances the detection rates of HSIL and cervical cancer. Furthermore, the results from TCT and HPV testing hold predictive value for postoperative pathological outcomes.^[19]^ This paper reports a postoperative pathological positivity rate of 67.6% among patients who tested positive for both the TCT and HPV. In this double-positive group, the incidence of postoperative pathological findings with a severity of ≥HSIL was significantly higher than in those who were not double positive (*P* < .05). This suggests that individuals testing positive for both TCT and HPV are more likely to exhibit ≥HSIL in postoperative pathology results, corroborating Jia study findings.^[20]^ Additionally, literature indicates that in postmenopausal patients with double positivity for TCT and HPV, diagnostic conization of the cervix is recommended even when biopsy results suggest LSIL or lower, to preclude any missed HSIL detection.^[21]^ In this research, the presence or absence of HPV did not significantly affect postoperative pathology results (*P* > .05), aligning with Zhu research.^[22]^ However, our study identifies a significant impact of HPV types on postoperative pathology (*P* < .05). Specifically, the ratio of postoperative pathology showing ≥HSIL was 0.551 times higher in cases with HPV16 or a combination of HPV16 with other types compared to cases with negative HPV or other types detected. This underscores that the probability of ≥HSIL in postoperative pathology is mainly increased in patients with HPV16, due to its greater pathogenicity compared to other types, supporting previous findings.^[23]^ Therefore, combined TCT and HPV screening is crucial in postmenopausal women. It is essential not to rely solely on the subjective judgment of the attending physician for screening decisions. In cases where postmenopausal individuals test positive for both TCT and HPV, particularly when HPV16 is present, proactive measures such as colposcopic cervical biopsy and ECC should be promptly initiated. For those who are double positive, a more active consideration of surgical interventions is recommended.

### 4.3. Characteristics of colposcopic assessment of HSIL in postmenopausal women

Decreased estrogen levels can impact the accuracy of colposcopic evaluations and tissue biopsies performed under colposcopy.^[24]^ This study found that factors such as colposcopic exposure, the squamocolumnar junction, and colposcopic interpretation did not have statistically significant effects on postoperative pathological outcomes (*P* > .05). This suggests that the extent of colposcopic exposure and the condition of the squamocolumnar junction do not influence the results of postoperative pathological assessments. These findings align with those reported in Fan’s study,^[25]^ which indicated that colposcopic manifestations do not significantly correlate with postoperative pathological outcomes in postmenopausal women. This lack of correlation is attributed to hormonal changes occurring during the postmenopausal period. Colposcopy interpretation did not significantly affect postoperative pathological outcomes, which may be associated with the expertise level and qualifications of colposcopy practitioners. However, this study demonstrates that the TZ significantly influences postoperative pathology (*P* < .05), with the probability of postoperative pathology classified as ≥HSIL increasing by 49.7% for each unit elevation in TZ. This finding is consistent with reports suggesting that individuals with TZ3 are more likely to exhibit advanced postoperative pathology.^[26]^ Research indicates that a reduced visibility of the complete cervical transformation zone correlates with a lower rate of pathological concordance between biopsy and LEEP.^[27]^ In cases where the cervical transformation area is classified as type 3, which often extends deep into the cervical canal and presents a narrower focus, focused lesion removal is advisable. Under these circumstances, considering CKC becomes increasingly pertinent. Previous studies recommend cervical resective treatment for cases identified as TZ3 during colposcopy.^[28]^ However, this recommendation requires validation with larger sample sizes to strengthen its credibility, providing valuable insights for future research. These findings suggest that TZ3 represents an independent risk factor for HSIL in postmenopausal women, advocating CKC as the preferred surgical approach for patients diagnosed with TZ3.

### 4.4. Choice of optimal surgical modality for postmenopausal women with HSIL

Currently, CKC is favored as the preferred treatment for HSIL due to its minimal surgical trauma, lower complication rates, faster postoperative recovery, and the achievement of both diagnostic and therapeutic goals.^[29–32]^ However, for postmenopausal patients, the selection of surgical modalities is more flexible owing to decreased estrogen levels, prevalent cervical atrophy, disappearance of the vaginal vault, and the absence of reproductive concerns. Nevertheless, in contemporary clinical practice, the primary consideration for postmenopausal patients involves choosing surgical methods that ensure minimal trauma, low rates of positive margins, cost-effectiveness, fewer complications, avoidance of psychological distress from repeated treatments, and prevention of secondary harm to the patients.

In our study, no cases (0%) were upgraded to SCC in the LEEP group, whereas there were 5 cases (3.1%) in the CKC group and 4 cases (3.7%) in the ETH group. This difference was statistically significant (*P* < .05). The absence of SCC upgrades in the LEEP group could be attributed to the selection of patients with mild cervical atrophy, favorable colposcopic conditions, and predominance of transformation zones 1 and 2 (TZ1-2). Moreover, our study reveals that the post-LEEP pathological margin positivity was 17.5%, indicating that despite favorable cervical conditions, the margin positivity rate following LEEP remains notably high. This high margin positivity serves as a significant risk factor for residual or recurrent postoperative lesions and is a crucial indicator for assessing patient prognosis.^[33]^ These findings suggest that LEEP may not be the first choice for postmenopausal patients, aligning with the conclusions of Liu study.^[34]^ Although LEEP offers benefits such as speed, minimal intraoperative bleeding, cost-effectiveness, and outpatient feasibility, the presence of positive margins presents challenges. Subsequent surgical interventions not only increase psychological burden on patients but also contribute to higher overall costs. Additionally, for some patients, undergoing direct CKC might prevent the need for a second operation, especially when extensive resection during CKC ensures negative margins, thus alleviating associated difficulties and potential adverse outcomes.

In this study, 9 cases of cervical cancer were unexpectedly identified, including 4 cases of stage IA1 and 1 case of stage IA2 in the CKC group, and a mixture of 4 cases across stages IA1, IA2, and IB1 in the ETH group. Due to its broader surgical scope, ETH increases the risk of collateral damage and associated costs. Moreover, some patients underwent total hysterectomy, which can complicate subsequent operations and potentially damage critical structures like the urinary system and blood vessels. According to the National Comprehensive Cancer Network’s clinical guidelines on cervical cancer, unexpected discovery of cervical cancer following total hysterectomy may require additional interventions, such as further surgery or radiotherapy.^[35]^ Therefore, for patients diagnosed with cervical cancer beyond stage IAI following ETH, pelvic external irradiation, and concurrent cisplatin chemotherapy were administered after thorough imaging evaluations and detailed discussions between the doctor and patient. However, this approach escalates costs and subjects patients to the side effects and complications of radiotherapy and chemotherapy, thereby increasing their psychological distress and physical discomfort. Additionally, some studies suggest potential risks of overtreatment and heightened complication rates associated with ETH for HSIL.^[36]^ There is also a significant recurrence rate after surgical interventions. These findings highlight that ETH may not be the optimal initial choice for postmenopausal women with HSIL. Consequently, patients initially treated with ETH require careful and comprehensive evaluations to prevent the need for additional surgical or radiotherapeutic interventions when cervical cancer progresses beyond stage IAI post-surgery.

This study reveals that the rate of postoperative pathology outcomes following CKC is 8.379 times higher than that of LEEP, and the likelihood of postoperative pathology ≥HSIL after ETH is 4.427 times higher than after LEEP. These findings suggest that both CKC and ETH are more effective than LEEP in patients with ≥HSIL, offering a significant advantage in reducing the risk of underdiagnosis due to limited surgical scope. The results also indicate that the rate of CKC margin positivity is lower than that of LEEP, which helps patients avoid the necessity for secondary surgery due to positive surgical margins. In cases where cervical cancer is detected through CKC, the majority are typically at stage IA1 or IA2. Consequently, staging surgery for cervical cancer becomes a viable option, potentially avoiding the discomfort and side effects associated with radiotherapy. Therefore, CKC offers distinct advantages over ETH in this context. Some studies suggest that for patients with HSIL on colposcopic biopsy, cervical conization, which can serve both diagnostic and therapeutic purposes, is preferable due to its ease of operation and minimal trauma. This aligns with the conclusions of this study.^[37]^ In summary, CKC should be considered the treatment of choice for postmenopausal HSIL. Compared to ETH, CKC avoids the risks of overtreatment and the complexities of secondary surgeries due to inadequate surgical scope. Moreover, CKC has safety advantages and a lower lesion residual rate compared to LEEP, making it a preferable method for treating HSIL in postmenopausal women with cervical atrophy.

## 5. Conclusions

1. Combined TCT and HPV screening is crucial for cervical cancer prevention, early detection, and management in postmenopausal women. Women with positive results for both TCT and HPV should undergo colposcopic cervical biopsy and endocervical curettage. 2. For patients with TZ3, CKC is the recommended surgical option. 3. CKC is the preferred treatment for postmenopausal women with HSIL, as it effectively diagnoses and treats the lesion, showing superior outcomes in managing postmenopausal HSIL.

## Acknowledgments

The authors want to thank the Shufang Song for making this research feasible. Thank you also to all the healthcare providers who have participated and who have shown tremendous engagement in providing care for women.

## Author contributions

**Conceptualization:** Shufang Song.

**Data curation:** Xiaofeng Zhao, Rong Zhang, Yu Wang, Xiaojie Mu.

**Formal analysis:** Xiaofeng Zhao, Rong Zhang, Yu Wang, Xiaojie Mu.

**Funding acquisition:** Shufang Song.

**Investigation:** Xiaofeng Zhao, Rong Zhang, Yu Wang.

**Project administration:** Shufang Song.

**Supervision:** Xiaojie Mu.

**Writing – original draft:** Xiaofeng Zhao.

**Writing – review & editing:** Xiaofeng Zhao, Yu Wang.
